# Supplementary material for: Learning curve and surgical outcome of robotic assisted colorectal surgery with ERAS program
Source: Sci Rep. 2022 Nov 29;12:20566. doi: 10.1038/s41598-022-24665-w (PMC9709162; doi:10.1038/s41598-022-24665-w)
Supplement: Supplementary file 1 — Supplementary Information 1. [file 41598_2022_24665_MOESM1_ESM.zip › LSC Raw data-Ñ[▒K/074/N_ñw╝╨░O▒Kñσ.pdf]

W102 / W102

臺中榮民總醫院
手術室護理記錄

[redacted]

基本資料

- 室別： 二醫手術室-31室，手術科別： CRS，手術類別： 常規，日期： 2020/03/25
- 進入時間： 1130 ； 麻醉開始時間： 1135 ； Time Out時間： 1230 ； 劃刀時間： 1231 ； 手術結束時間： 1655 送出時間： 1700 ；
- 麻醉方式： GE

  診斷： Malignant tumor of rectum

  手術名稱： 1.Low anterior resection with colo-anal anastomosis2.Ileostomy3.Laparoscopy

  手術局部用藥： nil

  術前預防性抗生素：
    - 抗生素名稱一： Veterin 1GM IVD(由麻醉科護理人員給予)
    - 時間： 1225
    - 上次簽章: 吳雅華

  ，追加預防性抗生素：
- 手術醫師： 蔣鋒帆，楊曙榮，陳映菁
- 麻醉醫師： VS 賴慧卿
-
    - 刷手護士A ： 戴宛榛，時間： 1130-1230，接替刷手一： 吳雅華，時間： 1230-1300，接替刷手二： 戴宛榛，時間： 1300
    - 流動護士A ： 吳雅華，時間： 1130-1200，接替流動一： 梁凱雯，時間： 1200-1300，接替流動二： 吳雅華，時間： 1300-1400
- 更多接替及流動護士： 接替刷手三： 王毓均，時間： 1500，接替流動三： 羅渝軒，時間： 1400

皮膚狀況

A-V Shunt： 無

術前： 不完整

- 3.擦傷

部位(術前)：

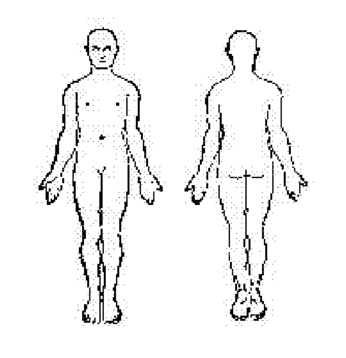

左手前臂及上臂

記錄者： 上次簽章: 吳雅華

術後： 不完整

- 3.擦傷

部位(術後)：

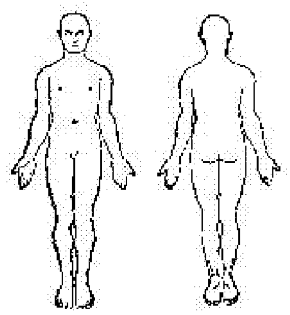

同術前

記錄者： 羅渝軒

手術準備

手術臥式 單一手術臥式

-
    - 手術臥式： 截石臥式
    - 使用附件： 矽膠枕頭，腳架
    - 皮膚準備： 10%Aq Betadine，Hibiscrub，其它： 2% Cjlorhexidine
    - 電燒： 使用電燒: 電極片位置： 其它： 迴路電極板，記錄者： 上次簽章: 吳雅華

止血帶T： 無

植入物

- 植入物名稱/號碼/數量/部位： SurgiWrap 0.02*100*130mm/壹片/in Abdomal

, 記錄者： 羅渝軒

標本送檢

- 細菌培養 無
- 細胞學 無
- 冰凍切片 無
- 病理標本
  組織名稱一： Distal Cut End
  ,
  組織名稱二： IMA Root
  ,
  組織名稱三： Rectum
- 截肢 無

標本交付 標本： ? 袋，標本： 零 盒，標本： 零 瓶，細胞學： 零 管，Culture Tube： 零 管，被交付醫師： CR 楊曙榮，

- 記錄者： 羅渝軒
- 時間： 1630

傷口術後敷料

傷口敷料： 傷口敷料： 無

- 請於圖上標示部位：

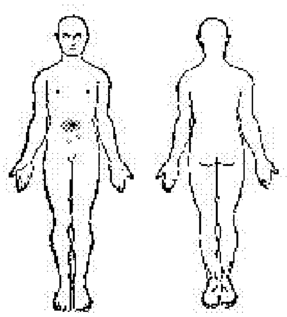

腹部

- 傷口敷料： Mepore貼覆固定

導管

導管	天數	導管資訊	插入日期	插入時間	更換日期	拔除日期	拔除時間	拔除原因	預計拔除/更換	預計拔除/更換日期
Sump NG Tube(1)	1	號碼:16 Fr.; 部位:胃; 註記:FIX 57CM;	2020/03/25	1255						
Foley(1)	1	號碼:16 Fr.; 部位:膀胱;	2020/03/25	1150						
Rod,Stoma Supporting (1)	1	部位:迴腸;	2020/03/25	1625						
A-line(1)	1	號碼:20 G; 部位:前臂; 象限:LU;	2020/03/25	1200						
Penrose Drain(1)	1	號碼:12 mm.; 部位:骨盆腔;	2020/03/25	1610						
CVC(1)	1	號碼:8 Fr.; 部位:; 象限:RU; 註記:fix 15cm;	2020/03/25	1210						

用物計數

- 手術中出血量： 100 c.c.
- 手術中尿量： 1300 c.c.
-
  - 其它： 無

用物計數： 多切口同時縫合

- 1.器械： 正確，2.縫針： 正確，3.刀片： 正確，4.紗布： 正確，5.腹部墊： 未使用，6.紗球： 未使用，7.PEANUTS ： 未使用，8.紗條： 未使用，9.絲帶： 未使用，10.針頭： 未使用，11.Vessel loop： 未使用，12.Boots： 未使用，13.Jelco： 未使用，14.棉條： 未使用，
  - 第一次：
    - 刷手護士 ： 戴宛榛
    - 流動護士： 羅渝軒
    - 時間： 1450

  ,
  - 第二次：
    - 刷手護士 ： 王毓均
    - 流動護士： 羅渝軒
    - 時間： 1550

  ,
  - 第三次：
    - 刷手護士 ： 王毓均
    - 流動護士： 羅渝軒
    - 時間： 1620

備註

備註： NIL

- 記錄者： 羅渝軒
- 時間： 1700

填寫護理人員 羅渝軒

正式摘要

W102 / W102

doctorRank:B
